# Prolactin and Estradiol are Epigenetic Modulators in Bovine Mammary Epithelial Cells during *Staphylococcus aureus* Infection

**DOI:** 10.3390/pathogens9070520

**Published:** 2020-06-28

**Authors:** María Guadalupe Salgado-Lora, Ivan Medina-Estrada, Joel Edmundo López-Meza, Alejandra Ochoa-Zarzosa

**Affiliations:** 1 Centro Multidisciplinario de Estudios en Biotecnología, Facultad de Medicina Veterinaria y Zootecnia, Universidad Michoacana de San Nicolás de Hidalgo, Morelia 58893, Mexico; rosa_imperial@hotmail.com (M.G.S.-L.); elmeza@umich.mx (J.E.L.-M.); 2 Trayectoria en Genómica Alimentaria, Universidad de la Ciénega del Estado de Michoacán de Ocampo, Sahuayo 59103, Mexico; rimedina@ucienegam.edu.mx

**Keywords:** mammary epithelial cells, prolactin, estradiol, innate immunity, epigenetic marks, *Staphylococcus aureus*

## Abstract

Changes in the levels of reproductive hormones compromise the bovine innate immune response (IIR). Changes in 17β-estradiol (E2) and prolactin (bPRL) levels affect the IIR of bovine mammary epithelial cells (bMECs), the target tissue of these hormones. In this work, we explored the effect of the combined hormones on bMEC IIR during *Staphylococcus aureus* infection, and if they can modulate epigenetic marks. By gentamicin protection assays, we determined that combined hormones (bPRL (5 ng/mL) and E2 (50 pg/mL)] decrease *S. aureus* internalization into bMECs (~50%), which was associated with a reduction in integrin α5β1 membrane abundance (MA) (~80%) determined by flow cytometry. Additionally, combined hormones increased Toll-like receptor 2 (TLR2) MA (~25%). By RT-qPCR, we showed that combined hormones induce the expression of pro- and anti-inflammatory cytokine genes, as well as up-regulate antimicrobial peptide gene expression. The combined hormones induced H3K9Ac at 12 h of treatment, which coincides with the reduction in histone deacetylase (HDAC, ~15%) activity. In addition, hormones increased the H3K9me2 mark at 12 h, which correlates with a reduction in the expression of KDM4A. In conclusion, bPRL and E2 modulate the IIR of bMECs, an effect that can be related to the regulation of histone H3 modifications such as H3K9Ac and H3K9me2.

## 1. Introduction

The innate immune response (IIR) is the first line of defense against infection, which involves the activation of different pathways through the recognition of pathogen-associated molecular patterns (PAMPs) by different pattern recognition receptors (PRRs). This response is strongly regulated at different levels, including the epigenetic mechanisms.

Mammary gland epithelial cells are targets of different reproductive hormones, such as prolactin (PRL) and estradiol (E2). These hormones regulate the proliferation, differentiation and survival of the mammary epithelium. Additionally, they can modulate the IIR of this tissue; however, the epigenetic role of these hormones in this response is little known [1,2]. In this sense, PRL and E2 inflammatory functions are diverse in several physiologic and pathologic conditions [3,4]. In mammary glands, these hormones can act alone or in combination, regulating the immune microenvironment [5]. In bovines, during the period around parturition, there is an increased susceptibility to inflammatory disorders in the mammary gland, such as mastitis [6]. This susceptibility has been correlated with abrupt hormonal changes, for example, E2 levels rise suddenly in the last week before parturition, increase in the last 3 days before delivery and fall rapidly after calving at basal values [6,7]. On the other hand, PRL also increases at calving, triggering lactogenesis and galactopoeisis [8]. In addition, E2 can potentiate the effects of others hormones on the mammary epithelium, for example, E2 with PRL stimulate proliferation more than either hormone alone and promote the progression of the epithelial structures toward a more differentiated state [9,10].

Bovine mammary epithelial cells (bMECs) play a relevant role during intramammary infections because they are in close contact with the bacteria responsible of mastitis, becoming a target for persistent bacteria causing chronic and subclinical infections, such as *Staphylococcus aureus* [11,12]. In addition, bMECs are able to orchestrate a relevant defense against infection [13]. The role of bovine PRL (bPRL) and E2 in the susceptibility of bMECs during *S. aureus* infection has been explored in our group evaluating either hormone alone, showing that bPRL at physiological concentrations (5 ng/mL) induces the internalization of *S. aureus* into bovine mammary epithelial cells, whereas E2 (50 pg/mL) reduces it. Both effects are achieved through the modulation of elements of the IIR of bMECs, such as cytokines and antimicrobial peptide production [14,15]. However, we do not know if the combination of these hormones, resembling an in vivo condition, could improve the defense response of bMECs. In addition, the epigenetic modulation of bMECs during *S. aureus* infection has been explored both in vitro as well in vivo. In this sense, Modak et al. [16], have reported that in a mice model for *S. aureus*-induced mastitis, bacteria induced hyperacetylation at histone H3K9 and H3K14 residues in mammary tissue. These acetylation marks were enriched at the promoters of overexpressed proinflammatory genes. Furthermore, Kweh et al. [17], have showed that the expression of β-defensins in bMECs is under the epigenetic control of DNA methylation and histone acetylation. In addition, the inhibition of histone deacetylases (HDACs) in bMECs inhibits the expression of inflammatory genes [18].

In spite of the evidence indicating an immunomodulatory role of hormones during *S. aureus* infection in bMECs, as well as the evidence showing the epigenetic regulation of inflammatory genes during mastitis, there are no studies related to the epigenetic hormonal modulation of the mammary epithelium during infection. Thus, the objective of this work was to analyze the effects of combined bPRL and E2 on bMEC defense during *S. aureus* infection and to determine if they can induce epigenetic modifications.

## 2. Results

### 2.1. The Combined Hormones Do Not Affect S. aureus nor bMEC Viability

**Figure 1:**
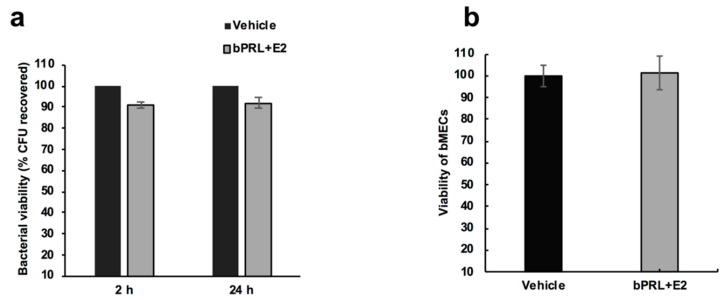
*S. aureus* growth and bovine mammary epithelial cell viability in the presence of the combined hormones. (**a**) Bacterial growth was determined counting the colony forming units (CFU)/mL of *S. aureus* treated with bovine prolactin (bPRL, 5 ng/mL) and 17β-estradiol (E2, 50 pg/mL) at 2 or 24 h. Each bar shows the mean of triplicates ± SE of three independent experiments (n = 9). The vehicle corresponds to bacteria treated with 1% ethanol. The effect of the vehicle was considered as 100% viability, and the effect of the hormones was normalized with respect to this control. (**b**) Bovine mammary epithelial cells (bMECs) were cultured with the combined hormones for 24 h and viability was calculated by a trypan blue exclusion assay. The number of viable bMECs is shown. Each bar shows the mean of triplicates ± SE of three independent experiments (n = 9). The effect of the vehicle was considered as 100% viability (1% ethanol).

With the purpose of analyzing if the combined hormones could affect the viability of *S. aureus*, we carried out a viability assay, incubating the bacteria with both hormones, and then counting colony forming units (CFUs) in Luria-Bertani agar (LBA) plates. The data showed that the combined hormone treatment (2 or 24 h) did not affect the bacterial viability (Figure 1a). Additionally, the viability of bMECs incubated with the combined hormones was evaluated by a trypan blue exclusion assay. According to the data shown in Figure 1b, we did not detect modifications in the number of viable bMECs in response to the hormonal treatment.

### 2.2. Internalization of S. aureus into bMECs is Reduced in Response to the Combined Hormones

**Figure 2:**
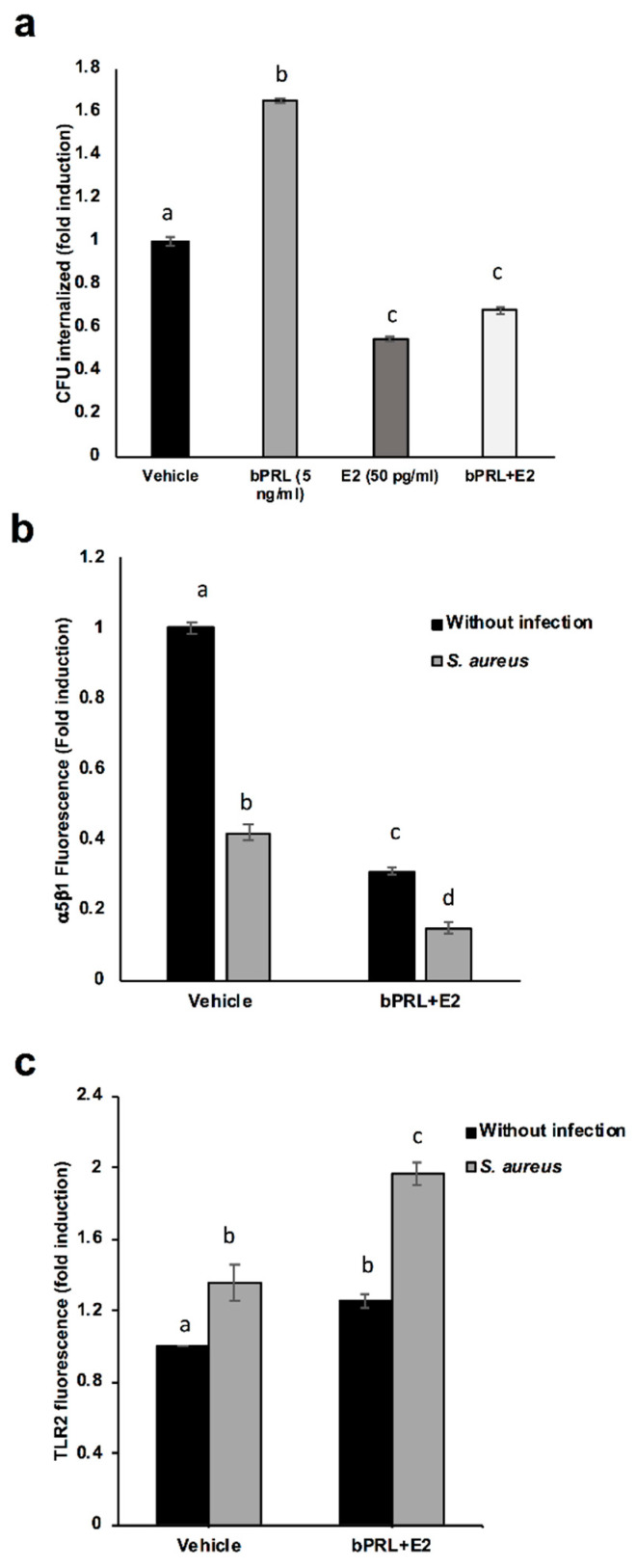
Effect of the combined hormones on *S. aureus* internalization: the role of α5β1 integrin and Toll-like receptor 2 (TLR2). (**a**) bMECs were treated with the combined hormones or left untreated (24), and then were challenged with *S. aureus* for 2 h, and after that were washed three times with PBS and incubated with gentamicin to eliminate extracellular bacteria. Data are shown as the percentage of CFU/mL recovered after bMEC lysis. Values were determined considering the control (bMECs cultured with the vehicle 1% ethanol) as 1. Each bar shows the mean of triplicates ± SE of three independent experiments (n = 9). (**b**) The relative fluorescence intensity of α5β1 membrane abundance in bMECs treated with the combined hormones and infected with *S. aureus* is shown. Fluorescence intensity was estimated from 10,000 events. The cells were fixed and stained extracellularly with an anti-α5β1 antibody and analyzed by flow cytometry. Each bar shows the mean of the fluorescence of 10,000 events ± SE, considering the fluorescence of control bMECs as 1 (data normalized). Data were obtained from two different experiments, which were run in triplicate (n = 6). (**c**) The relative fluorescence intensity of TLR2 membrane abundance in bMECs treated with the combined hormones and infected with *S. aureus* is shown. Fluorescence intensity was estimated from 10,000 events. The cells were fixed and stained extracellularly with an anti-TLR2 antibody and analyzed with flow cytometry. Each bar shows the mean of the fluorescence of 10,000 events ± SE, considering the fluorescence of control bMECs as 1 (data normalized). Data were obtained from two different experiments, which were run in triplicate (n = 6). Different letters between each condition analyzed indicate significant differences (one-way ANOVA, post hoc Tukey test *p* ≤ 0.05,) within the treatments.

We have previously shown that bPRL and E2 differentially regulate the internalization of *S. aureus* into bMECs, as bPRL induces it while E2 reduces bacterial invasion [15]. It is noteworthy that the combined hormones reduced the internalization of *S. aureus* into bMECs in a similar way to E2 alone (~40% of inhibition) (Figure 2a). With the purpose of explaining the mechanisms underlying this inhibition, we have previously reported that the receptor required for *S. aureus* internalization, the integrin α5β1, is down-regulated in the membrane upon infection (in a time-dependent fashion), presumably due to the internalization of the integrin bound to the bacteria [19]. In this work, we detected that the combined hormones reduce the integrin α5β1 MA before infection (~70% of inhibition), which could explain the reduction in the internalization shown in Figure 2a. As expected, in the presence of the bacteria, the reduction in the integrin α5β1 MA was higher (~85% of inhibition) (Figure 2b). The reduction in internalization is not due to the lack of recognition of *S. aureus* by bMECs, because the MA of TLR2 (the main innate response receptor that recognizes *S. aureus*) was enhanced in the presence of the hormones with relation to control bMECs (Figure 2c), which was maintained upon infection. This result indicates that the combination of bPRL and E2 could stimulate the IIR of bMECs during infection.

### 2.3. Expression of Pro-Inflammatory Genes in bMECs Treated with bPRL and E2

With the purpose of analyzing if the combined hormones modulate the IIR of bMECs, the expression of different pro-inflammatory, anti-inflammatory and antimicrobial peptides genes were analyzed. RT-qPCR analysis shown in Table 2 indicates that the combined hormones modulate IIR elements differentially: with respect to pro-inflammatory cytokines, *S. aureus* induced the expression of TNF-α, IL-1β and IL-6, as previously shown [15,19], the combined hormones increased the expression of TNF-α and IL-1β with respect to the control, but in infected bMECs, the expression of IL-1β (Table 1) was reduced in relation to infection alone. This result suggests that the combined hormones favor a pro-inflammatory environment in bMECs. We also detected that CXCL8 is up-regulated in bMECs pretreated with combined hormones and infected with *S. aureus* (~sixfold, Table 1).

### 2.4. Expression of Anti-Inflammatory and Antimicrobial Peptides Genes in bMECs Treated with bPRL and E2

We have reported that bMECs treated with E2 show an anti-inflammatory and antimicrobial response. In this study, the combined hormones induced the gene expression of the anti-inflammatory cytokine IL-10 (~twofold, Table 2), which was maintained as up-regulated upon infection. With relation to the expression of antimicrobial peptides, four different genes belonging to the defensin family of antimicrobial peptides were analyzed. The combined hormones induced the expression of BNBD10, LAP and DEFB1, which were maintained after infection (Table 2). Altogether, these results indicate that the combined hormones, besides the pro-inflammatory response described above, also can induce anti-inflammatory and antimicrobial responses in bMECs.

### 2.5. Regulation of H3 Acetylation by bPRL and E2 in bMECs during Infection

**Figure 3:**
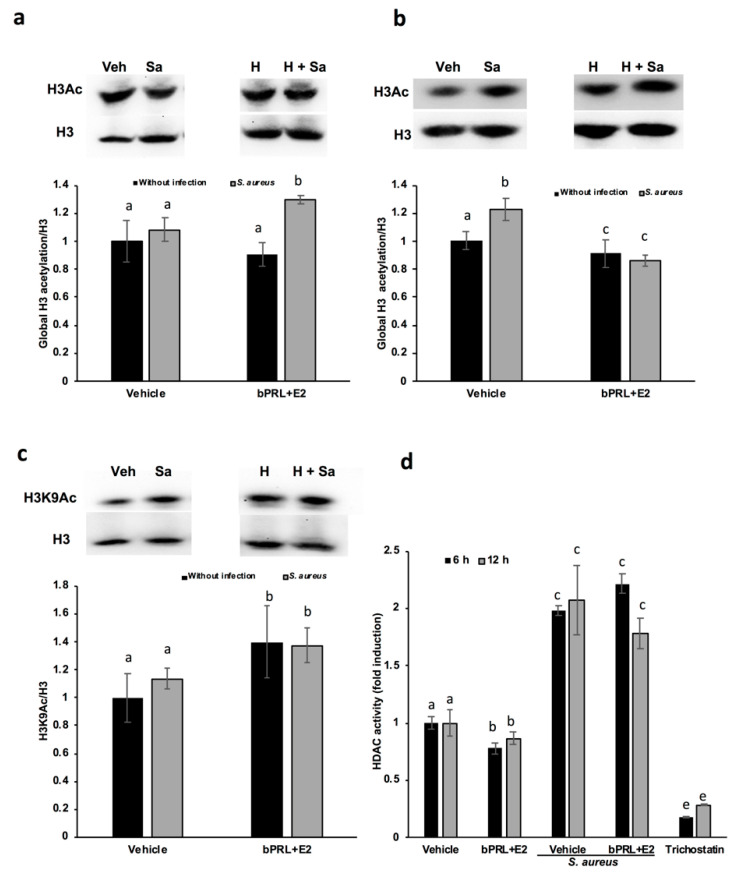
Regulation of H3 acetylation by bPRL and E2 in bMECs during infection. Densitometrical analysis of the immunoblots that shows the relative expression of H3Ac with respect total H3 in bMECs treated for 12 h (**a**) or 24 h (**b**) with the combined hormones. Additionally, representative western blot analysis is shown. In (**c**), the densitometrical analysis of the immunoblots that shows the relative expression of H3K9Ac with respect total H3 in bMECs treated for 12 h with the combined hormones is shown with its respective representative western blot analysis. Each bar shows the mean ± SE of optical density (arbitrary units, AU), considering the expression of control cells (1% ethanol) as 1 (data normalized), from four different experiments (n = 4). (**d**) bMEC lysates were obtained at 6 or 12 h of treatment with combined hormones with or without 2 h of *S. aureus* infection, and were incubated with the substrate for class I and II histone deacetylases (HDACs) accordingly to the manufacturer’s instructions. Total HDAC enzyme activity was determined by using the HDAC fluorometric cellular activity assay Fluor de Lys. Each bar shows the mean of HDAC activity of cells from two different experiments ± SE, which were run in duplicate (n = 4), considering the expression of control cells (1% ethanol) as 1 (data normalized). Different letters indicate significant differences between each condition (one-way ANOVA, post hoc Tukey test *p* ≤ 0.05). Trichostatin (1 μM) was employed as an inhibitor of HDACs. Veh: vehicle; Sa: *S. aureus*; H: combined hormones.

In order to explore if the effects of the combined hormones exerted on the IIR elements of bMECs could be related to epigenetic modulation, the state of histone acetylation was analyzed. The results shown in Figure 3a indicate that the combined hormones in the presence of bacteria increase the global acetylation of histone 3 (H3Ac, ~30%) at 12 h of treatment. However, at 24 h, the H3Ac mark was reduced to lower levels than the control in bMECs pretreated with the hormones with or without infection (Figure 3b). With the aim of determining the specific H3 residues acetylated, we analyzed the H3K9Ac mark, since it is induced by *S. aureus* infection in mice [16]. The results showed that combined hormones induce H3K9Ac at 12 h of treatment, both without and with infection (~40%, Figure 3c). Interestingly, at 24 h of hormonal treatment, there was no change in H3K9Ac (data not shown). To explore if the combined hormones could modulate the H3K9Ac mark through the regulation of the activity of HDACs, the activity of different class I and class II HDACs was analyzed. Evaluations were carried out at 6 and 12 h, considering that the effects on the H3K9Ac mark were detected at 12 h of hormonal treatment. According to the results, the combined hormones reduced the activity of HDACs both at 6 as well as 12 h of treatment (~23% and 15%, respectively, Figure 3d). The *S. aureus* infection increased HDAC activity (~100%), but in pretreated bMECs with the hormones, there were no significant changes in the enzymatic activity with respect to *S. aureus* infection alone (Figure 3d). In addition, the expression of the gene HDAC1 was analyzed in order to determine if this enzyme was responsible for these effects, but we did not detect changes in the level of its expression between treatments (data not shown).

### 2.6. Regulation of H3 Methylation by bPRL and E2 in bMECs during Infection

**Figure 4:**
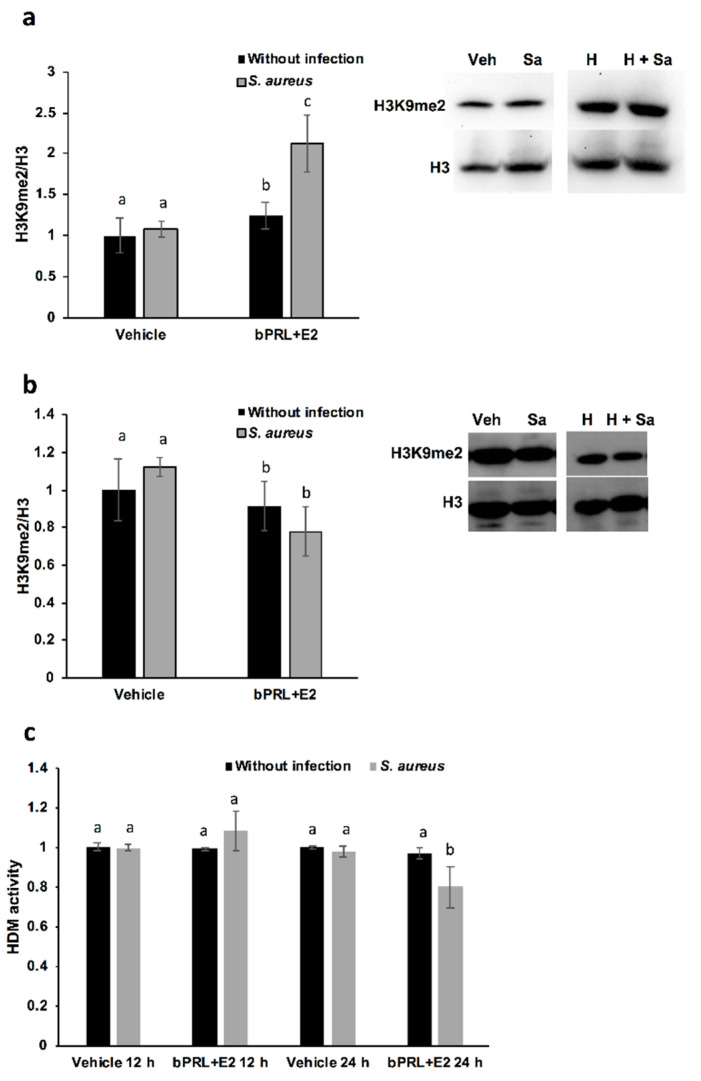
Regulation of H3 methylation by bPRL and E2 in bMECs during infection. Densitometrical analysis of the immunoblots that shows the relative expression of H3K9me2 with respect to total H3 in bMECs treated for 12 h (**a**) or 24 h (**b**) with the combined hormones. In addition, representative western blot analysis is shown. Each bar shows the mean ± SE of optical density (arbitrary units, AU), considering the expression of control cells (1% ethanol) as 1 (data normalized), from four different experiments (n = 4). (**c**) bMEC lysates were obtained at 24 h of treatment with combined hormones with or without 2 h of *S. aureus* infection, and were incubated with the substrates for Jumonji demethylases (HDMs). Each bar shows the mean of the HDM activity of cell lysates from two different experiments ± SE, which were run in duplicate (n = 4), considering the expression of control cells (1% ethanol) as 1 (data normalized). Different letters indicate significant differences between each condition (one-way ANOVA, post hoc Tukey test *p* ≤ 0.05. Veh: vehicle; Sa: *S. aureus*; H: combined hormones.

With the purpose of analyzing other epigenetic marks, such as methylation, we explored the regulation of dimethylation in H3K9 by the combined hormones in bMECs. According to the results, the combined hormones induced the H3K9me2 mark at 12 h of treatment (~20%, Figure 4a), and this effect was enhanced upon infection (~100%). However, at 24 h of treatment, this effect was reverted after 2 h of *S. aureus* infection, reducing the presence of H3K9me2 in bMECs (~23%, Figure 4b). This effect could be related to the reduction in histone demethylase activity detected in bMECs treated for 24 h with the hormones and infected with *S. aureus* (Figure 4c). At 12 h of treatment, we did not detect changes in histone demethylase (HDM) activity. Finally, the expression of the genes coding for the histone demethylases KDM4A and KDMAC (which are H3K9 histone demethylases) were analyzed. According to the results shown in Table 3, there is a reduction in the expression of KDM4A in bMECs treated for 12 h with the combined hormones and infection. These data could explain the enhanced H3K9me2 detected under the same conditions (Figure 4a).

## 3. Discussion

Cattle are prone to develop intramammary infections (IMIs) during the lactating cycle of the mammary gland. Although a higher susceptibility to IMIs occurs during the transition period (period from late pregnancy until early lactation), which is commonly defined as the period from 3 weeks before to 3 weeks after calving, IMIs can be also detected in non-lactating dairy cows [20,21]. This susceptibility has been correlated with hormonal changes, for example, E2 levels rise abruptly in the last week before parturition, and bPRL also increases at calving, triggering lactation [8]. In our group, we have previously explored the role of bPRL and E2 (separately) on the IIR of the bMECs, the target cells of these hormones and the host of intracellular *S. aureus.* bPRL induces *S. aureus* internalization into bMECs [19], but E2 reduces it [15], with both effects at physiological concentrations of the hormones. Since both hormones act together on bMECs in vivo, in this work, we explore the regulation of the elements of the IIR of these cells by the combination of hormones bPRL and E2 during *S. aureus* infection. Furthermore, we analyze if these effects could be related to epigenetic modulation.

First, we determined that the combined hormones neither significantly affected the viability of bacteria nor altered bMEC proliferation. These results agree with previous reports from our group [14,15]. On the other hand, combined hormones reduced *S. aureus* internalization into bMECs (35%), in a similar way to E2 [15]. In order to explain this reduction, the α5β1 integrin MA was evaluated, as it is the host receptor during *S. aureus* internalization in different epithelial cells. The α5β1 MA of bMECs was reduced by 70% in cells treated for 24 h with combined hormones (Figure 2b). The reduction in the MA observed in this receptor upon infection correlates with a previous report from our group, indicating that the internalization of bacteria results in the endocytosis of this receptor [19]. In this work, the α5β1 integrin MA was evaluated in cells infected for 2 h; however, we previously determined a reduction in the MA of this receptor after 15 min of infection [19]. Thus, the reduction in bacterial internalization could be related to the reduced MA of integrin α5β1 in hormone pre-treated bMECs.

With the purpose of determining if the combined hormones modulate the IIR of bMECs, the MA of TLR2 was analyzed, as this receptor is one of the main innate immune receptors recognizing *S. aureus* [22]. We have demonstrated that *S. aureus* induces TLR2 MA and activation in bMECs, and with respect to the hormones, bPRL stimulates TRL2 MA, whereas E2 reduces it [15,19]. In this work, combined hormones increased TLR2 MA (~25%), which was enhanced by infection (~100%). This result indicates that the combined hormones could favor the activation of the IIR of bMECs. Similar reports for the combination of bPRL and E2 have not been described in mammary glands; however, the role of these hormones in the immune microenvironment of mammary glands has been proposed [5]. In agreement with the activation of TLR2 induced by the combined hormones, we detected that the cytokine profile of bMECs stimulated by bPRL and E2 is mainly pro-inflammatory: hormones induce the expression of TNF-α and IL-1β, but also induce the expression of IL-10. This profile is maintained during infection, with the exception of IL-β, which is reduced in the presence of *S. aureus*. In addition, hormones up-regulate the expression of CXCL8 in infected bMECs. This effect is different from that reported previously for bPRL and E2 added separately to bMECs [15,19]. In particular, the up-regulation in CXCL8 expression is a remarkable effect of the combined hormones during infection, and it would be interesting to analyze in further research if leukocyte chemotaxis is induced under these conditions. According to previous work, the cytokine gene expression correlates with protein production [15]; thus, it is necessary in future experiments to perform protein determination in order to corroborate this. In this work, we also demonstrate that the combined hormones induce the expression of different antimicrobial peptides, which is maintained after infection. This effect was not detected with hormones added separately, indicating that the hormonal combination could lead to a better defense against *S. aureus*. A synergism achieved by PRL and E2 on the expression of different genes has been reported in human mammary cells [23], but the cross-talking between PRL and E2 signaling pathways requires further research.

In this work, we attempt to correlate the effect of hormones on innate immune genes with epigenetic marks. The analysis of the epigenetic modulation was carried out for different durations (6, 12 and 24 h) of hormonal stimulation, because the epigenetic effects on chromatin can be achieved with short durations [16], and the effects detected on gene expression at 24 h could be the consequence of immediate chromatin rearrangement. In this work, we analyzed post-translational modifications in histone H3K9, such as acetylation and dimethylation; additionally, the role of HDACs and HDMs in the abovementioned epigenetic marks was evaluated. Our results indicate that the global acetylation of H3 increases in bMECs infected and treated for 12 h with the combined hormones. This mark was down-regulated at 24 h of hormonal treatment (Figure 3). The H3K9Ac could be related to the global H3Ac, considering that we detected an up-regulation in this mark at 12 h of hormonal treatment in bMECs before infection, but interestingly, this mark was maintained after 2 h of infection with *S. aureus*. In agreement, Modak et al. (2014) reported that this mark is induced by *S. aureus* in mouse mammary glands [16], using an in vivo mastitis model. In our model, we detected a subtle up-regulation in H3K9Ac induced by *S. aureus*, which was enhanced by the combined hormones. To our knowledge, this is the first report that evaluates the H3K9Ac mark in the bovine mammary epithelium in response to bPRL and E2, which could be related to the up-regulation detected in the inflammatory genes. In addition, this mark can be also related to the global H3Ac, because the antibody employed detects the acetylation in lysine residues K9, K14, K18, K23 and K27. In this sense, it is necessary to analyze the other marks, given that H3K14ac is up-regulated in mouse mammary glands upon *S. aureus* infection [16]. The H3K9Ac mark induced by the combined hormones at 12 h of treatment coincides with the reduction in the activity of HDACs at 6 and 12 h (Figure 4d). This reduction indicates that the histone acetylation is maintained because the enzymes that remove acetylation marks are down-regulated or inhibited. Accordingly, Kweh et al. (2019) have reported that HDACs participate in the regulation of the expression of antimicrobial peptides in primary bMECs [17]. In addition, Romanick et al. (2018) indicate that HDACs 1 and 2 regulate inflammatory gene expression via canonical and noncanonical mechanisms in the bovine mammary epithelial cell line MAC-T [18]. In this work, we also demonstrated that *S. aureus* induces HDAC activation in bMECs. To our knowledge, this is the first report that shows this activity induced by *S. aureus*. In addition, Silva et al. (2018) have shown that short chain fatty acids inhibited HDAC activity, whereas they increased H3Ac in MAC-T cells [24]. We observed a similar effect in this work with the combined hormones. The kit employed to analyze HDACs detects the activity of class I HDACs (HDAC1, 2, 3 and 8), IIa (HDAC4, 5, 7 and 9), and IIb (HDAC6 and 10). We did not detect any change in the levels of HDAC1 gene expression (data not shown), therefore other HDAC(s) should be implicated in the reduced enzymatic activity observed.

With respect to the epigenetic mark of H3K9me2, this is positively associated with hypoacetylation, therefore reducing DNA transcription [25]. In this work, we demonstrated that this mark is induced in bMECs treated for 12 h with the combined hormones and infected, but at 24 h this mark is down-regulated under the same conditions. This is the first report that shows the role of this epigenetic mark in the bovine mammary epithelium. It is interesting that this mark coincides with H3K9Ac, as the combined hormones induce it in infected bMECs at 12 h, but at 24 h it is repressed. Altogether, these results suggest that the combined hormones can regulate the gene expression of inflammatory mediators through different epigenetic marks. In order to corroborate which genes are turned on or turned off by hormones, it is necessary to perform chromatin immunoprecipitation (ChIP) assays.

On the other hand, with the purpose of corroborating if HDMs are related to H3K9me2, we measure Jumonji family demethylases. Interestingly, HDM activity is reduced in bMECs treated for 24 h with the combined hormones and infected with *S. aureus*, a result that coincides with the reduced H3K9me2 detected by a western blot under the same conditions. We attempt to correlate this activity with the levels of histone lysine demethylases (KDMs), in particular, with members of the family KDM4, which have high substrate preferences for lysine residues on histone H3. KDM4s can demethylate H3K9me2/3, H3K27me2/3 and H3K36me3 [26]. In bovines, the role of these enzymes has been studied, mainly in fertilized oocytes [27]. We detected a reduction in KDM4A gene expression at 12 h in bMECs treated with the combined hormones and infected, which could be responsible for reducing HDM activity under the same conditions. Finally, the epigenetic modulation of hormones in bMECs is interesting in terms of describing the novel effects of bPRL and E2. In this sense, the epigenetic modulation of PRL in bovine mammary glands has been mainly studied during mammary gland growth, development and differentiation, correlating epigenetic marks with milk protein production and differentiation [28,29]. Otherwise, studies related to the regulation of epigenetic marks in mammary glands by E2 have been mainly associated with the study of breast cancer [30]. Thus, this work described, for the first time, that bPRL and E2 could be epigenetic modulators in bMECs and *S. aureus* infection.

## 4. Materials and Methods

### 4.1. Reagents

Native purified bovine prolactin (bPRL) (lot AFP7170E) was provided by A. F. Parlow (NHPP, NIDDK, Torrance, CA, USA), dissolved in water and sterilized by filtration. Sigma-Aldrich (St. Louis, MO, USA) provided 17β-estradiol (E2), and working solutions were dissolved in 1% ethanol. For all of the experiments, 1% ethanol (vehicle) was used as a control. The hormone concentrations used in this work were bPRL 5 ng/mL and E2 50 pg/mL, as reported [14,15].

### 4.2. Antibodies

For flow cytometry analysis, the blocking antibody anti-α5β1 integrin (MAB2514) was purchased from Millipore (Burlington, MA, USA). The blocking anti-TLR2 (TL2.1) antibody was obtained from Abcam. Fluorescein isothiocyanate (FITC)-conjugated secondary antibodies against mouse or rat IgGs were purchased from Cell Signaling Technology.

For western blot analysis, the rabbit polyclonal antibody anti-H3Ac (Abcam, ab47915), the mouse monoclonal antibody anti-H3K9Ac (Santa Cruz, Sc56616), the mouse monoclonal antibody anti-H3K9me2 (Abcam, ab1220) and the rabbit polyclonal antibody anti-H3 (Abcam, ab1791) were used. Secondary antibodies raised against rabbit or mouse IgGs and coupled to horseradish peroxidase were obtained from Cell Signaling Technology (Danvers, MA, USA).

### 4.3. Staphylococcus aureus Strain

The *S. aureus* subsp. *aureus* (ATCC 27543) strain was used, which was isolated from a case of bovine clinical mastitis and has the capacity to invade bMECs [31]. Bacteria were grown at 37 °C overnight in Luria–Bertani broth (LB, Bioxon), and the CFUs were adjusted by measuring the optical density at 600 nm (OD 0.2 = 9.2 × 10^7^ CFU/mL).

### 4.4. Primary Culture of Bovine Mammary Epithelial Cells (bMECs)

bMECs were isolated from the alveolar tissue of the udders of healthy lactating cows (slaughtered for meat production), as described [19]. Cells from passages 2–8 were used in all of the experiments. bMECs were cultured in growth medium (GM) composed by DMEM medium/nutrient mixture F12 Ham (DMEM/F12K, Sigma) and supplemented with 10% fetal calf serum (Equitech Bio), 10 μg/mL insulin (Sigma), 5 μg/mL hydrocortisone (Sigma), 100 U/mL penicillin, 100 μg/mL streptomycin and 1 μg/mL amphotericin B (Invitrogen, Carlsbad, CA, USA). bMECs were grown in a 5% CO_2_ atmosphere at 37 °C. All of the experiments were achieved in DMEM/F12K without phenol red (Sigma).

### 4.5. bMEC Viability and S. aureus Growth Assays

To determine the effect of bPRL and E2 on bMEC viability, 1 × 10^4^ cells were incubated with the combined hormones (5 ng/mL of bPRL together with 50 pg/mL of E2) in an incomplete medium for 24 h at 37 °C in 96-well plates. The bMEC viability was tested using the trypan blue exclusion assay and the cells were counted in an automated cell counter (BIO-RAD, TC20).

To analyze the effect of the combined hormones on *S. aureus* growth, 9.2 × 10^7^ CFU/mL were cultured at 37 °C in LB broth and growth was evaluated turbidimetrically. To evaluate the *S. aureus* viability in the presence of the combined hormones, bacteria were grown (as previously described) in LB broth and were treated with the hormones for 2 or 24 h at 37 °C. Later, the bacteria were plated on LB agar in triplicate and were incubated overnight at 37 °C. The number of CFUs was determined by standard colony counting. Each experiment was performed three times in triplicate. The data represent the percentage of cell viability, considering the cells treated with vehicle as 100% (1% ethanol).

### 4.6. Invasion Assays

For the infection assays, bMECs were cultured in serum-free DMEM/F12K without phenol red (Sigma) and antibiotics (incomplete medium) for 24 h (~1 × 10^4^ cells were cultured onto 96-well flat-bottomed dishes (Corning, Corning, NY, USA) treated with 6–10 μg/cm^2^ rat-tail type I collagen (Sigma)), and then bMECs were incubated with the hormones and/or infected. For the hormonal treatment, 5 ng/mL of bPRL, together with 50 pg/mL of E2 were added to incomplete medium for 24 h and then were infected with *S. aureus* (MOI 30:1 bacteria per cell). For this, the bMECs were inoculated with 9.2 × 10^7^ CFU/mL and incubated for 2 h in 5% CO_2_ at 37 °C. Then, the cells were washed three times with PBS (pH 7.4) and incubated with incomplete medium that was supplemented with 80 μg/mL of gentamicin for 1 h at 37 °C to eliminate extracellular bacteria. Finally, the bMEC monolayers were detached with trypsin (0.05%)-EDTA (0.02%) (Sigma) and lysed with 250 μL of sterile distilled water. The bMEC lysates were diluted 100-fold, plated on LB agar in triplicate and incubated overnight at 37 °C. The number of CFUs was determined by the standard colony counting technique. The number of bMECs cultured in each well plate was calculated for each invasion assay using a hemocytometer. Each experiment was performed three times in triplicate. The data represent the normalized ratio of the CFU recovered per bMEC, considering the cells treated with vehicle as 1 (1% ethanol).

### 4.7. Analysis of Receptors by Flow Cytometry

The bMEC-polarized monolayers were cultured in 24-well dishes (Corning) and were treated with the combined hormones for 24 h and/or infected with *S. aureus*, as described above. After this, the cells were washed three times with PBS, detached with trypsin (0.05%)-EDTA (0.02%) (Sigma), centrifuged at 3200× *g* for 10 min at 4 °C and washed with PBS. The bMEC pellet was blocked with normal goat serum (5% in PBS, Pierce, Rockford, IL, USA) for 30 min at 4 °C with shaking, and then the cells were centrifuged and the pellet was incubated with the primary antibodies anti-TLR2 or anti-integrin, separately. To measure the TLR2 membrane abundance (MA), the cells were incubated with the antibody at a dilution of 1:50 (PBS containing BSA 0.1%) for 1 h at 4 °C with shaking. For the determination of integrin MA, the cells were incubated with the anti-α5β1 integrin at a concentration of 10 μg/mL for 2 h at 4 °C with shaking. In all of the cases, after the primary antibody incubation, the bMECs were washed three times with PBS and incubated with the respective secondary antibody (diluted 1:50) (FITC-conjugated anti-mouse IgGs for TLR2 or anti-rat IgGs for α5β1 integrin) for 1 h at 4 °C with shaking in the dark. The pellet was recovered by centrifugation, washed and fixed with paraformaldehyde (4%) for 10 min at 4 °C and finally washed three times with PBS. The cells were then suspended in 100 μL of PBS. The fluorescent signals of 10,000 events were measured and evaluated using the BD AccuriTM C6 cytometer, and analyzed with FlowJo software. Each experiment was performed three times in triplicate. The data represent the normalized receptor membrane abundance, considering the cells treated with vehicle as 1 (1% ethanol).

### 4.8. RNA Isolation and Gene Expression Analysis

To analyze the effects of the combined hormones and/or *S. aureus* on the expression of the IIR genes of bMECs, monolayers of cells cultured in six-well plates with 6–10 μg/cm^2^ rat-tail type I collagen (Sigma) were incubated with the hormones (24 h) and/or *S. aureus* for 2 h (MOI 30:1). The same procedure was performed to evaluate the genes of the epigenetic enzymes, but for these approaches, bMECs were incubated with hormones for 12 or 24 h, and/or *S. aureus* for 2 h. bMEC total RNA (5 µg) was extracted with the Trizol reagent (Invitrogen) according to the manufacturer’s instructions. Genomic DNA contamination was removed from RNA samples with DNase I treatment (Invitrogen). Then, cDNA was synthesized as described [19]. The relative quantification of gene expression (qPCR) was performed using the comparative Ct method (∆∆Ct) in a StepOne Plus Real-Time PCR System (Applied Biosystems, Foster City, CA, USA) according to the manufacturer’s instructions. The reactions were carried out with VeriQuest SYBR Green qPCR master mix (Affymetrix, Santa Clara, CA, USA). Specific primer pairs were acquired from Invitrogen and Elim Biopharm (Hayward, CA, USA) (Table 4), and their specificity was determined by end point PCR. The GAPDH gene was used as an internal control. Each experiment was performed four times in duplicate and the ∆∆Ct value was obtained by setting the basal expression of vehicle-treated bMECs as RQ = 1.

### 4.9. Western Blot Analysis

To analyze epigenetic marks, the histone extraction of bMECs was performed according to the protocol described by Shechter et al. [32] for the acid extraction of histones. Briefly, bMECs (3 × 10^6^ cells) were incubated in Petri dishes at confluence, then the treatments were added (the combined hormones for 12 or 24 h and/or *S. aureus* for 2 h). After this, the cells were washed three times with PBS, detached with trypsin (0.05%)-EDTA (0.02%) (Sigma), centrifuged at 3200× *g* for 10 min at 4 °C and washed with PBS. The bMEC pellet was resuspended in 1 mL hypotonic lysis buffer and transferred to a 1.5-mL tube. Then, it was incubated for 30 min on a rotator at 4 °C to promote the hypotonic swelling of cells and lysis by mechanical shearing during rotation. The intact nuclei were pelleted by spinning in a cooled tabletop centrifuge at 15,400× *g*, 10 min, 4 °C and were re-suspended in 400 μL 0.4 N H_2_SO_4_. To lysis nuclei, they were incubated on a rotator overnight and then were centrifuged to remove nuclear debris at 15,400× *g*, 10 min. The supernatant was incubated with TCA (final concentration of 33%) to precipitate the histones; then, they were incubated overnight at 4 °C. Histones were pelleted by spinning in a cooled tabletop centrifuge at 15,400× *g*, 10 min at 4 °C. Histone pellets were washed with ice-cold acetone, and centrifugated at 15,400× *g*, 5 min at 4 °C. This step was repeated three times. Then, histones were air-dried for 20 min at room temperature. Histone pellets were dissolved in ddH_2_O and stored frozen at −20 °C. Histones were resolved on a 15% SDS-PAGE gel to verify integrity and concentration before performing western blot analysis.

Proteins were transferred to PVDF membranes using a semi-dry transference system (Fisher Scientific); then were blocked with 5% non-fat milk in PBS for 4 h at 4 °C. After that, membranes were incubated with primary antibodies at 4 °C overnight: anti-H3Ac (1:1,000), anti-H3K9Ac (1:200), anti-H3K9me2 (1:1000) or the control antibody anti-H3 (1:5000). Membranes were incubated with anti-mouse or anti-rabbit horseradish peroxidase-coupled secondary antibodies (1:3000) for 2 h at 4 °C. Finally, ECL western blotting substrate WesternSureTM (Thermo Scientific, Waltham, MA, USA) was added and membranes were exposed to X-ray film or were developed using the iBright CL1500 Imaging System (Thermo Scientific). Signal intensity was quantified by densitometry using iBright Analysis Software (Thermo Scientific). Each western blot was performed at least three times from different experiments. The data represent the normalized ratio of the target histone mark with relation to H3 expression, considering the cells treated with vehicle as 1 (1% ethanol).

### 4.10. Histone Deacetylase (HDAC) Activity Assay

Plates of 96 wells were used to grow 1 × 10^5^ bMECs with the combined hormones for 6 or 12 h, and then were infected with *S. aureus* for 2 h. HDAC activity was measured using a Fluor de Lys^®^ kit (Enzo LifeSciences, Farmingdale, NY, USA) to analyze class I HDACs (HDAC1, 2, 3 and 8), IIa (HDAC4, 5, 7 and 9) and IIb (HDAC6 and 10). Then, bMECs were incubated with 2000 pmol of the acetylated Fluor de Lys^®^ substrate for 4 h. Cells, media and the standard curve of the deacetylated substrate were prepared according to the manufacturer’s directions; relative fluorescence unit (RFU) counts were acquired with a Varioskan (Enzo LifeSciences) plate reader (360 nm excitation, 460 nm emission). A standard curve was generated using serial 1:10 dilutions of the deacetylated Fluor de Lys standard and developer supplied with the BML-AK503 HDAC fluorometric cellular activity assay kit. Trichostatin (TSA, 1 μM) was purchased from Sigma, and was used as an inhibitor of HDACs. HDAC activity was calculated, considering the effect of vehicle-treated bMECs as basal activity (normalized to onefold). According to the manufacturer’s instructions, the assays were run in duplicate from two different experiments.

### 4.11. Histone Demethylase (HDM) Activity Assay

We employed a kit from ThermoFisher (Cat. EIAHDMF) (Waltham, MA, USA) to measure the activity of Jumonji family demethylases (HDMs) in bMECs treated with the combined hormones and infected. Cells were grown in plates of 96 wells and treated as described for the HDAC assay, and cell lysates were obtained according to the manufacturer’s instructions. The product of the enzymatic demethylation reactions is formaldehyde, which was quantitated directly by a fluorescent product with a Varioskan (Thermo Scientific) plate reader (450 nm excitation, 510 nm emission). HDM activity was calculated, considering the effect of vehicle-treated bMECs as basal activity (normalized to onefold). According to the manufacturer’s instructions, the assays were run in duplicate from two different experiments.

### 4.12. Statistical Analysis

The data were analyzed in PRISM 8.0 software by performing a one-way analysis of variance (one-way ANOVA) and using the post hoc Tukey test. The results are reported as the means ± the standard errors (SE), and the significance level was set at *p* ≤ 0.05, except for RT-qPCR analysis, where fold change values greater than two or less than 0.5 were considered as significantly differentially expressed mRNAs [33].

## 5. Conclusions

Altogether, these results indicate that bPRL and E2 are able to modulate the innate immune response of bMECs during *S. aureus* infection, favoring a pro-inflammatory and antimicrobial response, which results in diminishing bacterial internalization. All these effects could be related to the regulation of histone H3 modifications, such as H3K9Ac and H3K9me2.

## Figures and Tables

**Table 1:** Expression of inflammatory genes in bMECs treated with bPRL and E2 ^1^.

Pro-Inflammatory Gene	Treatments			
	Vehicle	*S. aureus*	E2 + bPRL	E2 + bPRL + *S. aureus*
**TNF-α**	1 ± 0.06	**2.18 ± 0.06**	**2. 99 ± 0.07**	**3.02 ± 0.09**
**IL-1β**	1 ± 0.04	**18.33 ± 0.77**	**3.1 ± 0.05**	**8.4 ± 0.26**
**IL-6**	1 ± 0.05	**2.12 ± 0.19**	1.56 ± 0.05	**3.18 ± 0.11**
**CXCL8**	1 ± 0.08	1.84 ± 0.15	0.88 ± 0.05	**5.83 ± 0.04**

^1^ RT-qPCR analysis where fold change values greater than two or less than 0.5 were considered as significantly differentially expressed mRNAs. Relative quantity (RQ) values are shown. Differentially expressed genes are indicated in bold.

**Table 2:** Expression of anti-inflammatory and antimicrobial peptide genes in bMECs treated with bPRL and E2 ^1^.

Anti-Inflammatory and Antimicrobial Genes	Treatments			
	Vehicle	S. *aureus*	E2 + bPRL	E2 + bPRL +*S. aureus*
**IL-10**	1 ± 0.06	**2.03 ± 0.01**	**2.23 ± 0.05**	**2.03 ± 0.01**
**BNBD10**	1 ± 0.04	1.26 ± 0.06	**7.63 ± 0.02**	**14.90 ± 0.03**
**TAP**	1 ± 0.05	0.58 ± 0.02	0.68 ± 0.03	0.65 ± 0.02
**LAP**	1 ± 0.04	0.92 ± 0.06	**2.08 ± 0.11**	**2.33 ± 0.04**
**DEFB1**	1 ± 0.09	2.22 ± 0.08	**2.22 ± 0.01**	**2.06 ± 0.04**

^1^ RT-qPCR analysis where fold change values greater than two or less than 0.5 were considered as significantly differentially expressed mRNAs. RQ values are shown. Differentially expressed genes are indicated in bold.

**Table 3:** Expression of demethylase genes in bMECs treated with bPRL and E2 ^1^.

Treatments	Gene KDM4A		Gene KDM4C	
	12 h	24 h	12 h	24 h
**Vehicle**	1 ± 0.01	1 ± 0.015	1 ± 0.06	1 ± 0.02
***S. aureus***	1.43 ± 0.02	0.96 ± 0.04	1.87 ± 0.08	0.73 ± 0.01
**E2 + PRL**	1.46 ± 0.04	0.79 ± 0.0	1.91 ± 0.1	1.32 ± 0.0
**E2 + PRL + *Sa***	**0.48 ± 0.01**	0.87 ± 0.03	1.06 ± 0.08	0.76 ± 0.07

^1^ RT-qPCR analysis where fold change values greater than two or less than 0.5 were considered as significantly differentially expressed mRNAs. RQ values are shown. Differentially expressed genes are indicated in bold. *Sa*: *S. aureus*.

**Table 4:** Oligonucleotides of inflammatory response and epigenetic genes used in this study.

Genes (bovine) ^1^	Primer	Sequence (5′→3′)	Fragment (bp)	Tm (°C)	Reference
BNBD10	F R	GCTCCATCACCTGCTCCTC AGGTGCCAATCTGTCTCATGA	152	54	[15]
DEFB1	F R	CCATCACCTGCTCCTCACA ACCTCCACCTGCAGCATT	185	54	[15]
GAPDH	F R	TCAACGGGAAGCTCACTGG CCCCAGCATCGAAGGTAGA	237	57	[15]
HDAC1	F R	TTACGACGGGGATGTTGGAA GGCTTTGTGAGGACCGTTGG	136	62	This work
IL-10	F R	GATGCGAGCACCCTGTCTGA GCTGTGCAGTTGGTCCTTCATT	129	59	[12]
IL-1α	F R	GCAGAAGGGAAGGGAAGAATGTAG CAGGCTGGCTTTGAGTGAGTAGAA	198	52	[15]
IL-6	F R	AACCACTCCAGCCACAAACACT GAATGCCCAGGAACTACCACAA	179	57	[12]
CXCL8	F R	TTCCACACCTTTCCACCCCAA GCACAACCTTCTGCACCCACTT	149	53.5	[12]
KDM4A	F R	CAGAAATGTGCTTCACCTCG CACCGAGAACACATCCAGTC	178	58	This work
KDM4C	F R	GGAACACCCGGTACTACAG CACTTGACTTGCACGACTTC	154	62	This work
LAP	F R	GCCAGCATGAGGCTCCATC CTCCTGCAGCATTTTACTTGGGCT	194	54	[15]
TAP	F R	GCGCTCCTCTTCCTGGTCCTG GCACGTTCTGACTGGGCATTGA	216	57	[15]
TNF-α	F R	CCCCTGGAGATAACCTCCCA CAGACGGGAGACAGGAGAGC	101	56	[12]

^1^ BNDB10: Bovine neutrophil beta defensin 10; DEFB1: Defensin beta 1; GAPDH: Glyceraldehyde 3-phosphate dehydrogenase; HDAC1: Histone deacetylase 1; IL-10: Interleukin-10; IL-1β: Interleukin 1-β; IL-6: Interleukin-6; CXCL8: Chemokine interleukin-8; KDM4A: Lysine demethylase 4A; KDM4C: Lysine demethylase 4C; LAP: Lingual antimicrobial peptide; TAP: Tracheal antimicrobial peptide; TNF-α: Tumor necrosis factor α.

## Abbreviations

bMECs	Bovine mammary epithelial cells
bPRL	Bovine prolactin
IIR	Innate immune response
E2	17β-estradiol
HDACs	Histone deacetylases
HDMs	Histone demethylases
H3K9Ac	Acetylation of lysine 9 of H3
H3K9me2	Dimethylation of lysine 9 of H3

## References

[1] Lamote I., Meyer E., De Ketelaere A., Duchateau L., Burvenich C. (2006). Influence of sex steroids on the viability and CD11b, CD18 and CD47 expression of blood neutrophils from dairy cows in the last month of gestation. Vet. Res..

[2] Sackmann-Sala L., Guidotti J.E., Goffin V. (2015). Minireview: Prolactin regulation of adult stem cells. Mol. Endocrinol..

[3] Pereira Suarez A.L., López-Rincón G., Martínez Neri P.A., Estrada-Chávez C. (2015). Prolactin in inflammatory response. Adv. Exp. Med. Biol..

[4] Kovats S. (2015). Estrogen receptors regulate innate immune cells and signaling pathways. Cell. Immunol..

[5] Need E.F., Atashgaran V., Ingman W.V., Dasari P. (2014). Hormonal regulation of the immune microenvironment in the mammary gland. J. Mammary Gland Biol. Neoplasia.

[6] Lamote I., Meyer E., De Ketelaere A., Duchateau L., Burvenich C. (2006). Expression of the estrogen receptor in blood neutrophils of dairy cows during the periparturient period. Theriogenology.

[7] Akers R.M. (2006). Major advances associated with hormone and growth factor regulation of mammary growth and lactation in dairy cows. J. Dairy Sci..

[8] Vanacker N., Ollier S., Beaudoin F., Blouin R., Lacasse P. (2017). Effect of inhibiting the lactogenic signal at calving on milk production and metabolic and immune perturbations in dairy cows. J. Dairy Sci..

[9] Berryhill G.E., Trott J.F., Hovey R.C. (2016). Mammary gland development—It’s not just about estrogen. J. Dairy Sci..

[10] Horigan K.C., Trott J.F., Barndollar A.S., Scudder J.M., Blauwiekel R.M., Hovey R.C. (2009). Hormone interactions confer specific proliferative and histomorphogenic responses in the porcine mammary gland. Domest. Anim. Endocrinol..

[11] Benić M., Maćešić N., Cvetnić L., Habrun B., Cvetnić Z., Turk R., Đuričić D., Lojkić M., Dobranić V., Valpotić H. (2018). Bovine mastitis: A persistent and evolving problem requiring novel approaches for its control—A review. Vet. Arh..

[12] Alva-Murillo N., López-Meza J.E., Ochoa-Zarzosa A. (2014). Nonprofessional phagocytic cell receptors involved in *Staphylococcus aureus* internalization. Biomed. Res. Int..

[13] Günther J., Seyfert H.M. (2018). The first line of defence: Insights into mechanisms and relevance of phagocytosis in epithelial cells. Seminars in Immunopathology.

[14] Lara-Zárate L., López-Meza J.E., Ochoa-Zarzosa A. (2011). *Staphylococcus aureus* inhibits nuclear factor kappa B activation mediated by prolactin in bovine mammary epithelial cells. Microb. Pathog..

[15] Medina-Estrada I., López-Meza J.E., Ochoa-Zarzosa A. (2016). Anti-inflammatory and antimicrobial effects of estradiol in bovine mammary epithelial cells during *Staphylococcus aureus* internalization. Mediat. Inflamm..

[16] Modak R., Das Mitra S., Vasudevan M., Krishnamoorthy P., Kumar M., Bhat A.V., Bhuvana M., Ghosh S.K., Shome B.R., Kundu T.K. (2014). Epigenetic response in mice mastitis: Role of histone H3 acetylation and microRNA(s) in the regulation of host inflammatory gene expression during *Staphylococcus aureus* infection. Clin. Epigenet..

[17] Kweh M.F., Merriman K.E., Nelson C.D. (2019). Short communication: Inhibition of DNA methyltransferase and histone deacetylase increases β-defensin expression but not the effects of lipopolysaccharide or 1,25-dihydroxyvitamin D. J. Dairy Sci..

[18] Romanick S.S., Morrill K., Hostler A., Evans L.W., Shen Y., Matsumura A., Piotrowski H., Silva L.G., Faciola A.P., Ferguson B.S. (2019). HDAC1/2-mediated regulation of JNK and ERK phosphorylation in bovine mammary epithelial cells in response to TNF-α. J. Cell. Physiol..

[19] Medina-Estrada I., Alva-Murillo N., López-Meza J.E., Ochoa-Zarzosa A. (2015). Non-classical effects of prolactin on the innate immune response of bovine mammary epithelial cells: Implications during *Staphylococcus aureus* internalization. Microb. Pathog..

[20] Enger B.D., Nickerson S.C., Tucker H.L.M., Parsons C.L.M., Akers R.M. (2019). Apoptosis and proliferation in *Staphylococcus aureus*-challenged, nonlactating mammary glands stimulated to grow rapidly and develop with estradiol and progesterone. J. Dairy Sci..

[21] Esposito G., Irons P.C., Webb E.C., Chapwanya A. (2014). Interactions between negative energy balance, metabolic diseases, uterine health and immune response in transition dairy cows. Anim. Reprod. Sci..

[22] Akira S., Uematsu S., Takeuchi O. (2006). Pathogen recognition and innate immunity. Cell.

[23] Rasmussen L.M., Frederiksen K.S., Din N., Galsgaard E., Christensen L., Berchtold M.W., Panina S. (2010). Prolactin and oestrogen synergistically regulate gene expression and proliferation of breast cancer cells. Endocr. Relat. Cancer.

[24] Silva L.G., Ferguson B.S., Avila A.S., Faciola A.P. (2018). Sodium propionate and sodium butyrate effects on histone deacetylase (HDAC) activity, histone acetylation, and inflammatory gene expression in bovine mammary epithelial cells. J. Anim. Sci..

[25] Liu W., Zhai Z., Chen L., Wang L., Zhang D., Ren W., Zhao Y., Shen Z. (2017). The global profile of histone H3 acetylation and methylation is affected by FOXP3. Int. J. Clin. Exp. Med..

[26] Lee D.H., Kim G.W., Jeon Y.H., Yoo J., Lee S.W., Kwon S.H. (2020). Advances in histone demethylase KDM4 as cancer therapeutic targets. FASEB J..

[27] Pagé-Larivière F., Sirard M.A. (2014). Spatiotemporal expression of DNA demethylation enzymes and histone demethylases in bovine embryos. Cell. Reprogram..

[28] Singh K., Erdman R.A., Swanson K.M., Molenaar A.J., Maqbool N.J., Wheeler T.T., Arias J.A., Quinn-Walsh E.C., Stelwagen K. (2010). Epigenetic regulation of milk production in dairy cows. J. Mammary Gland Biol. Neoplasia.

[29] Rijnkels M., Freeman-Zadrowski C., Hernandez J., Potluri V., Wang L., Li W., Lemay D.G. (2013). Epigenetic modifications unlock the milk protein gene loci during mouse mammary gland development and differentiation. PLoS ONE.

[30] Bhan A., Hussain I., Ansari K.I., Bobzean S.A., Perrotti L.I., Mandal S.S. (2014). Histone methyltransferase EZH2 is transcriptionally induced by estradiol as well as estrogenic endocrine disruptors bisphenol-A and diethylstilbestrol. J. Mol. Biol..

[31] Anaya-López J.L., Contreras-Guzmán O.E., Cárabez-Trejo A., Baizabal-Aguirre V.M., López-Meza J.E., Valdez-Alarcón J.J., Ochoa-Zarzosa A. (2006). Invasive potential of bacterial isolates associated with subclinical bovine mastitis. Res. Vet. Sci..

[32] Shechter D., Dormann H.L., Allis C.D., Hake S.B. (2007). Extraction, purification and analysis of histones. Nat. Protoc..

[33] Morey J.S., Ryan J.C., Van Dolah F.M. (2006). Microarray validation: Factors influencing correlation between oligonucleotide microarrays and real-time PCR. Biol. Proced. Online.

